# Supplementary material for: Genome-Wide Identification and Characterization of the Cyclophilin Gene Family in the Nematophagous Fungus Purpureocillium lilacinum
Source: Int J Mol Sci. 2019 Jun 18;20(12):2978. doi: 10.3390/ijms20122978 (PMC6627767; doi:10.3390/ijms20122978)
Supplement: Supplementary file 1 [file ijms-20-02978-s001.zip › proofread/Supplementary Material/2019.6.7 Supplementary figures.docx]

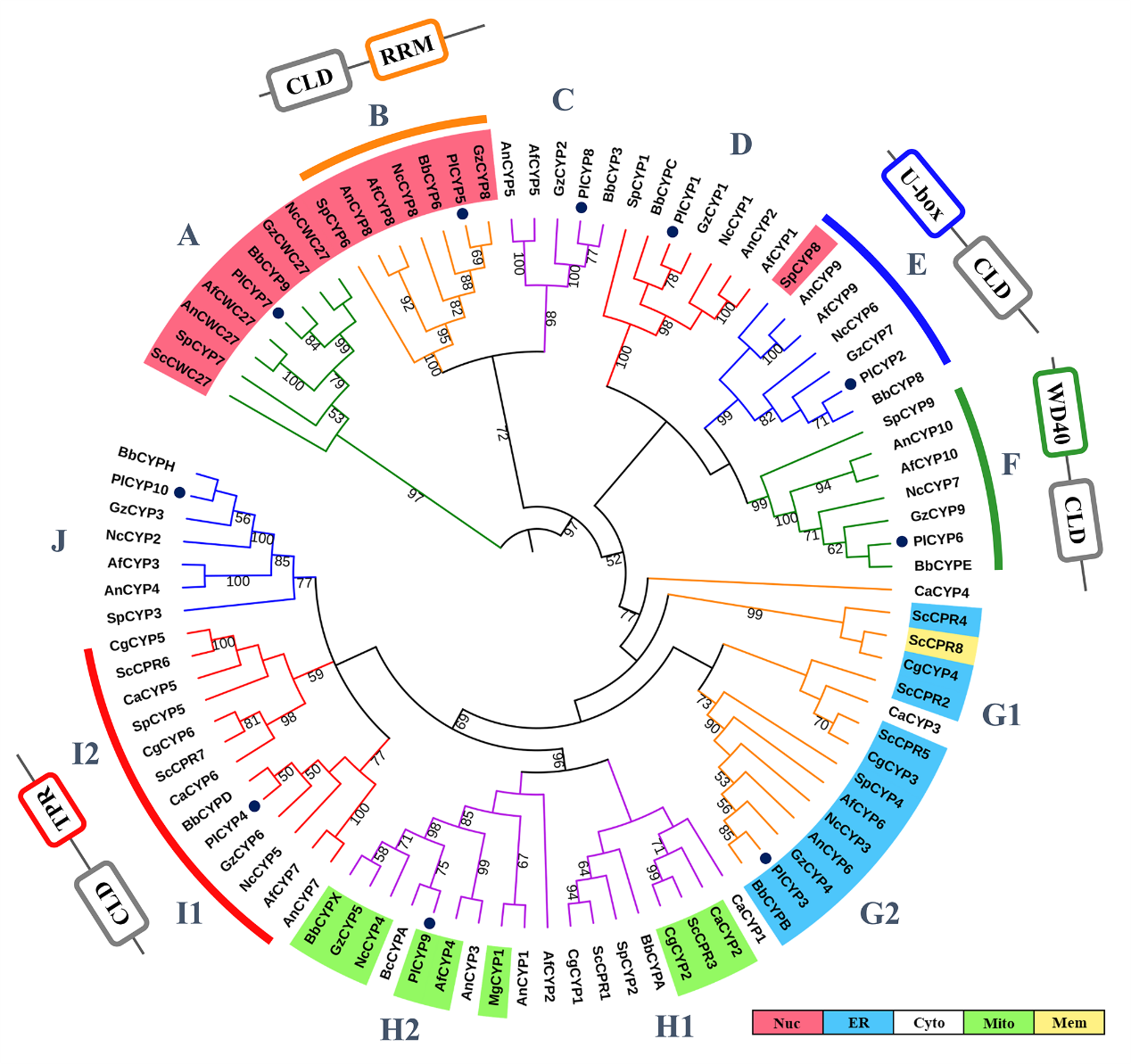


**Figure S1.** The phylogenetic tree for Ascomycota fungal CYP families. A total of 94 CYPs identified from *Purpureocillium lilacinum* (Pl), *Candida albicans* (Ca), *Candida glabrata* (Cg), *S. cerevisiae* (Sc), *Schizosaccharomyces pombe* (Sp), *Aspergillus fumigatus* (Af), *Aspergillus nidulans* (An), *Gibberella zeae* (Gz), *Neurospora crassa* (Nc), and *Beauveria bassiana* (Bb) were aligned by the MUSCLE program. The tree was generated by MEGA 6.0 using the Neighbor-Joining (NJ) method, with 1000 bootstrap replicates. The colored branches indicate different groups which are marked with letters (A to J). The predicted protein subcellular localizations were demonstrated by staining the label background. The positions of the PlCYPs in the tree are indicated by solid blue circles.


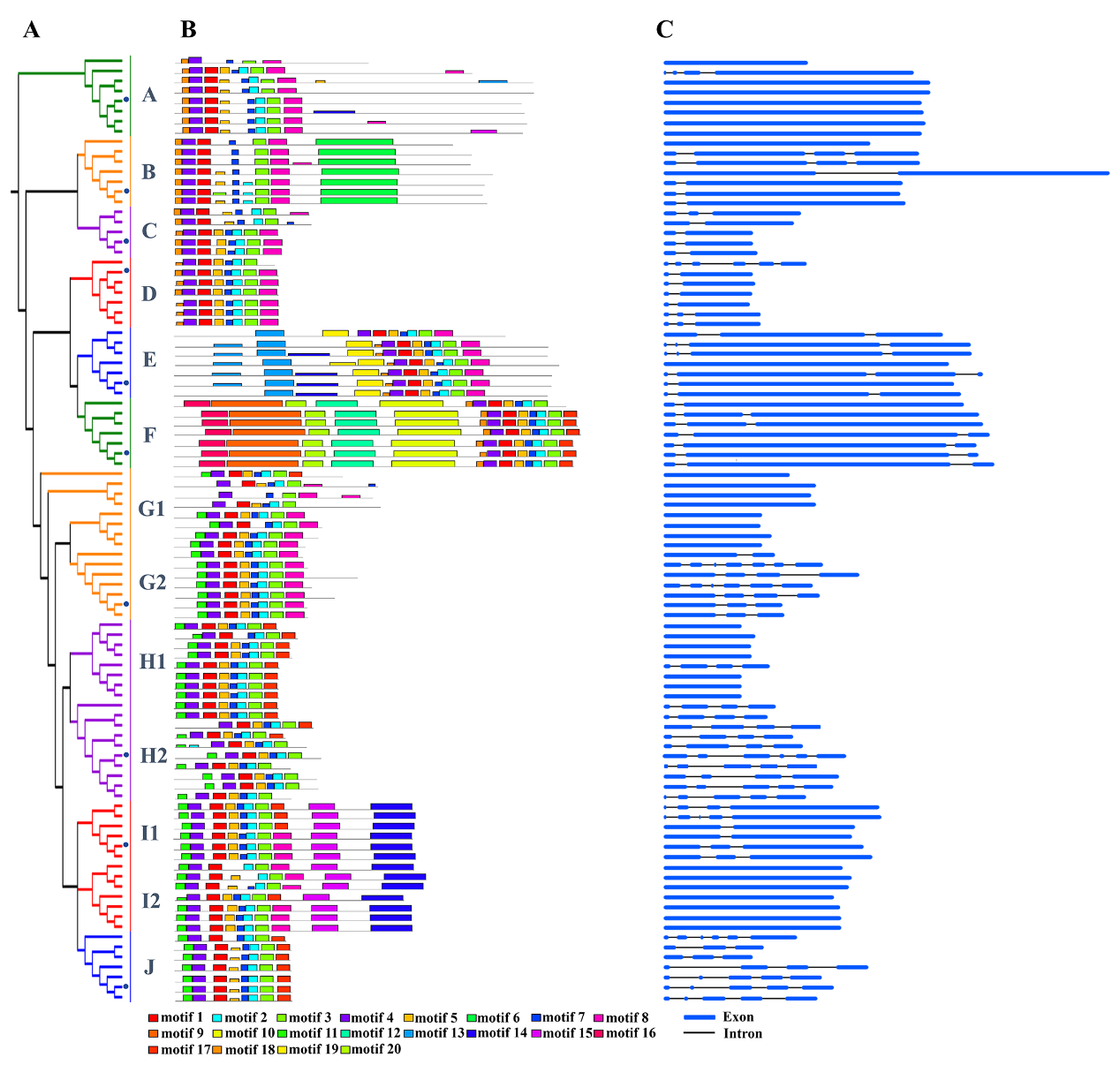


**Figure S2.** Motif and gene structure analyses of Ascomycota fungal CYPs. (A) Phylogenetic tree of the fungal CYPs. (B) Twenty motifs identified by the MEME program in different fungal CYPs. The motifs are highlighted with different colored blocks. (C) Gene structures of the fungal CYPs. Exons and introns are represented by blue boxes and black lines.


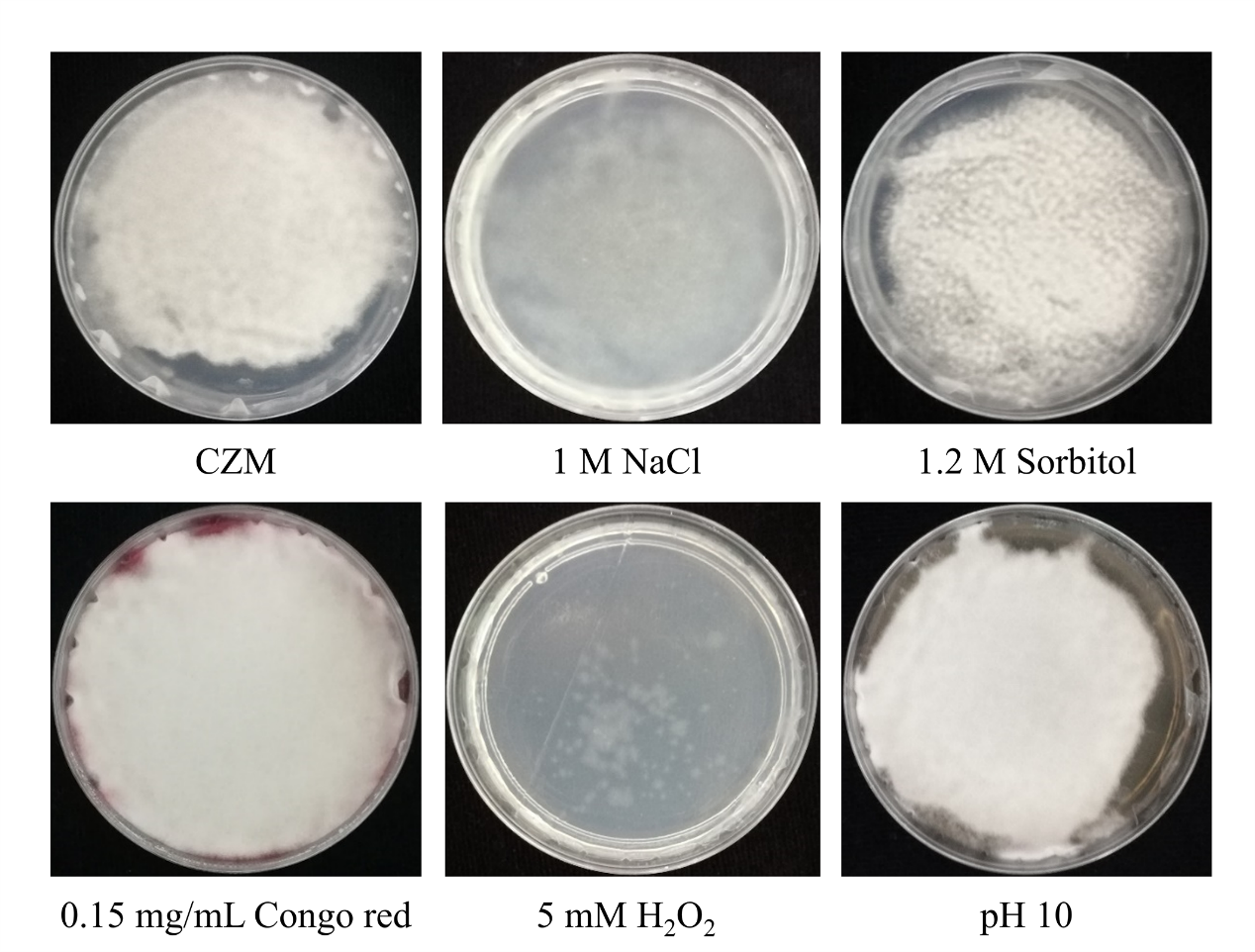


**Figure S3.** Growth of *P. lilacinum* strain 36-1 on CZM plates under abiotic stresses. 50 μL of *P. lilacinum* conidia standardized to 1×10^5^ conidia mL^-1^ were spread on normal CZM, CZM containing 1M NaCl, 1.2M sorbitol, 5mM H_2_O_2_ or 0.15 mg/mL Congo red, and CZM (pH 10). All plates were cultured at 28 ℃ for 3 days.


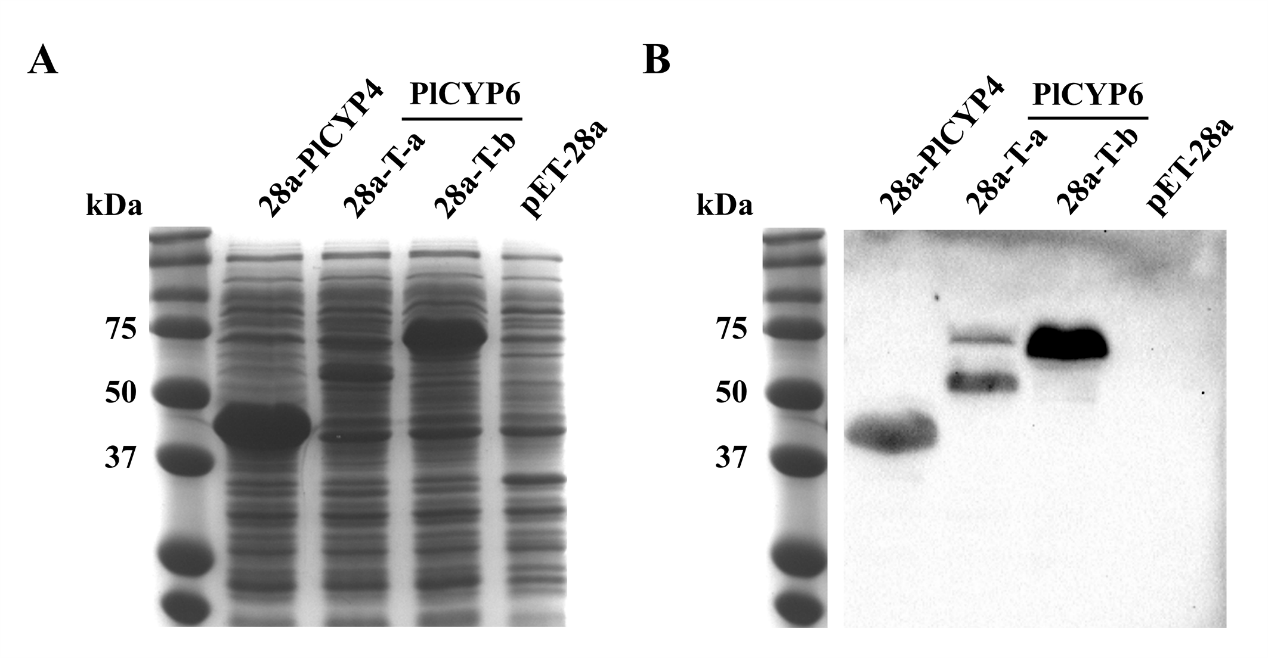


**Figure S4.** Inducible expressions of *PlCYP4* and *PlCYP6* in *E. coli*. (A) Protein separation by SDS-PAGE. (B) Result of hybridization with His label antibodies.


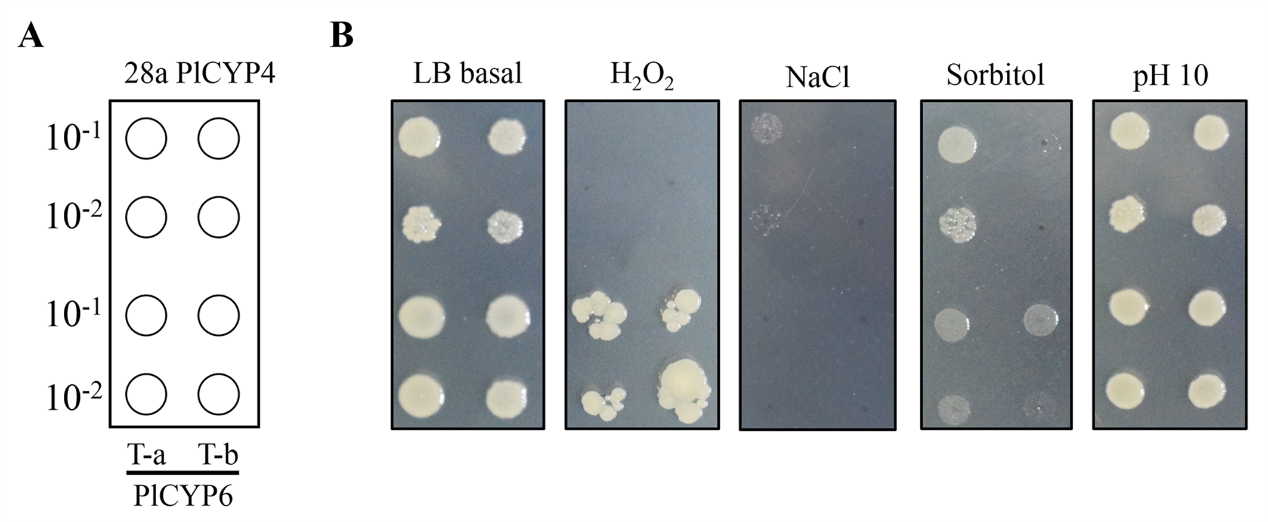


**Figure** **S5.** Growth of *E. coli* transformed with *PlCYP4* and *PlCYP6* genes towards abiotic stresses. (A) Diagram of spot assay. (B) Spot assay of *E. coli* cells containing empty vector (pET-28a) and recombinant vectors (pET-PlCYP4 and pET-PlCYP6) on LB plate supplemented with 1 mM H_2_O_2_, 600 mM NaCl, 800 mM Sorbitol, and LB medium that the pH was adjusted to ten. LB basal medium was used as control. 2 μL of each diluent was spotted on above mentioned medium, respectively.


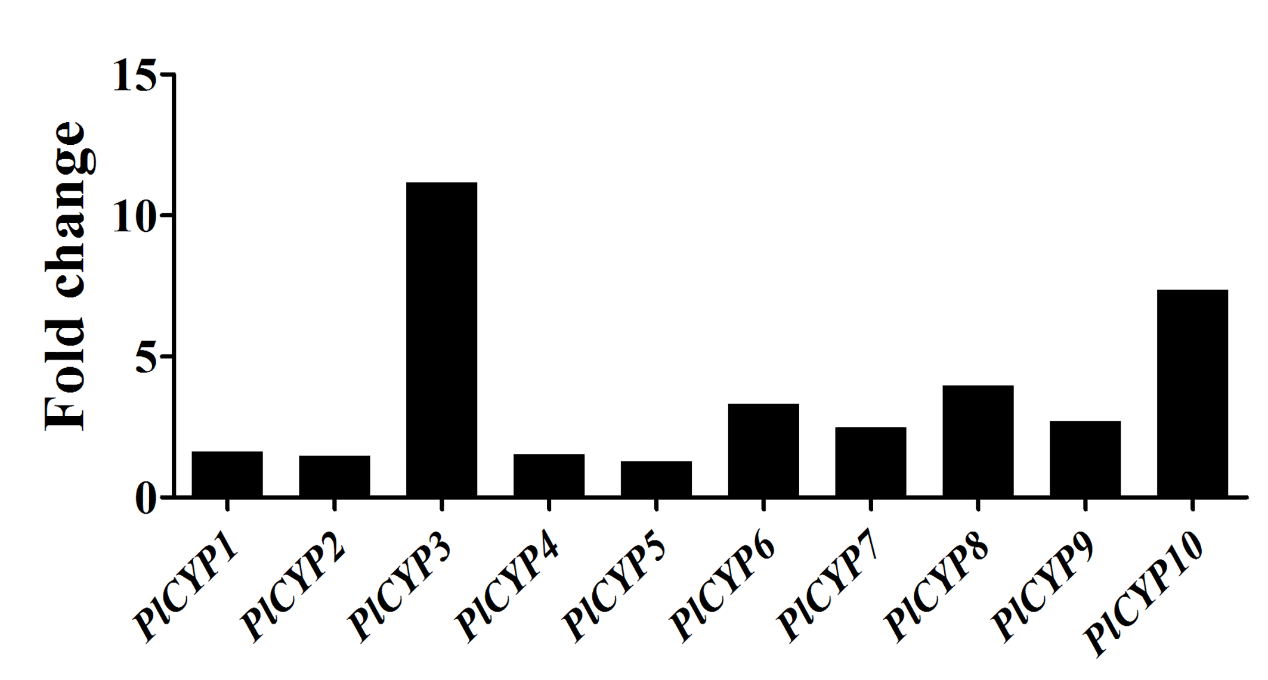


**Figure S6.** Expression levels of PlCYPs in the transcriptome data of *P. lilacinum* 36-1. The fold changes of gene expressions were calculated by the 2^-△△Ct^ method


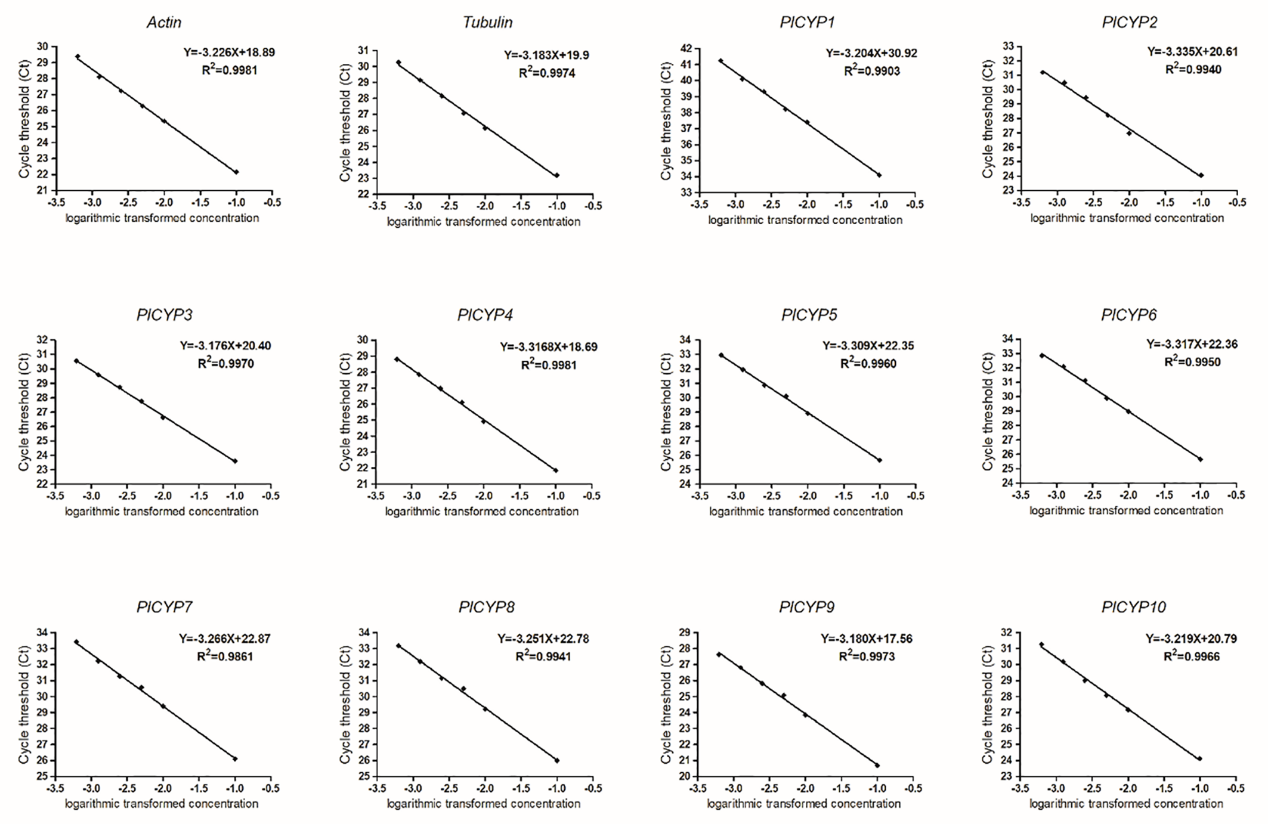


**Figure S7.** Amplification efficiencies of the primers used for qRT-PCR. The cDNA templates were obtained from reverse transcription of RNA extracted from *P. lilacinum* strain 36-1, and then were diluted 100, 200, 400, 800 and 1600 times. *y-axes* represented cycle threshold and *x-axes* represented the logarithmic transformed concertration of cDNA template. The matched curve equation was generated from the data.
