# Supplementary material for: Genome-Wide Identification and Characterization of the Cyclophilin Gene Family in the Nematophagous Fungus Purpureocillium lilacinum
Source: Int J Mol Sci. 2019 Jun 18;20(12):2978. doi: 10.3390/ijms20122978 (PMC6627767; doi:10.3390/ijms20122978)
Supplement: Supplementary file 1 [file ijms-20-02978-s001.zip › proofread/manuscript.v5.docx]

Article

Genome-Wide Identification and Characterization of the Cyclophilin Gene Family in the Nematophagous Fungus *Purpureocillium lilacinum*

Chenmi Mo, Chong Xie, Gaofeng Wang, Juan Liu, Qiuyan Hao, Xueqiong Xiao * and Yannong Xiao *

The Provincial Key Lab of Plant Pathology of Hubei Province, College of Plant Science and Technology, Huazhong Agricultural University, Wuhan, 430070, Hubei Province, PR China. [mochenmi@webmail.hzau.edu.cn](mailto:mochenmi@webmail.hzau.edu.cn) (C.M.); [xiechong@webmail.hzau.edu.cn](mailto:xiechong@webmail.hzau.edu.cn) (C.X.); [jksgo@mail.hzau.edu.cn](mailto:jksgo@mail.hzau.edu.cn) (G.W.); [juandalin0704@163.com](mailto:juandalin0704@163.com) (J.L.); [15738393165@163.com](mailto:15738393165@163.com) (Q.H.)

***** Correspondence: [xueqiongxiao@mail.hzau.edu.cn](mailto:xueqiongxiao@mail.hzau.edu.cn) (X.X.); [yannongxiao@mail.hzau.edu.cn](mailto:yannongxiao@mail.hzau.edu.cn) (Y.X.)

Received: 17 May 2019; Accepted: 13 June 2019; Published: date

**Abstract:** *Purpureocillium lilacinum* has been widely used as a commercial biocontrol agent for the control of plant parasitic nematodes. Whole genome analysis promotes the identification of functional genes and the exploration of their molecular mechanisms. The *Cyclophilin* (*CYP*) gene family belongs to the immunophillin superfamily, and has a conserved cyclophilin-like domain (CLD). CYPs are widely identified in prokaryotes and eukaryotes, and can be divided into single- and multi-domain proteins. In the present study, 10 *CYP* genes possessing the CLD, named *PlCYP1–P10*, were identified from the genome of *P. lilacinum* strain 36-1. Those 10 PlCYPs were predicted to have different cellular localizations in *P. lilacinum*. Phylogenetic and gene structure analysis revealed the evolutionary differentiation of CYPs between Ascomycotina and Saccharomycotina fungi, but conservation within the Ascomycotina fungi. Motif and gene structure distributions further support the result of phylogenetic analysis. Each *PlCYP* gene had a specific expression pattern in different development stages of *P. lilacinum* and its parasitism stage on eggs of *Meloidogyne incognita*. In addition, the 10 *PlCYP* genes exhibited different expression abundances in response to abiotic stresses, among which *PlCYP4* was highly expressed at a high temperature (35 °C), while *PlCYP6* was up-regulated under 5 mM of H_2_O_2_ stress. Furthermore, the heterologous expression of *PlCYP4* and *PlCYP6* in *Escherichia coli* enhanced the cellular tolerance against a high temperature and H_2_O_2_. In summary, our study indicates the potential functions of *PlCYPs* in virulence and the stress response, and also provides a frame for further analysis of the *CYP* gene family in Ascomycotina fungi.

**Keywords:** *Purpureocillium lilacinum;* cyclophilin; phylogenetic analysis; expression pattern; abiotic stress

1. Introduction

Plant parasitic nematodes infect almost all cultivated plants worldwide and cause huge economic losses of up to $157 billion per year [1]. It is difficult to discover nematode disease in fields owing to the small size of nematodes and the inconspicuous symptoms of infected plants [2]. The control of plant parasitic nematodes mainly depends on the application of nematicides, including carbofuran, ethoprophos, and aldicarb, while these pesticides have gradually been forbidden due to their adverse effects on the environment and human health. Therefore, biological control has become an alternative environment-friendly strategy for disease control. The nematophagous fungus, *Purpureocillium lilacinum*, is one of the most extensively tested fungi for controlling plant parasitic nematodes [3]. A number of studies have shown that *P. lilacinum* has a highly negative effect on the reproduction of nematodes. The application of *P. lilacinum* could effectively control second-stage juveniles, eggs, or egg masses of root-knot nematodes in pot experiments [4]. The combination of *Syncephalastrum racemosum* and *P. linacinum* can significantly decrease galls and nematodes in soil [5]. By soil application of *P. lilacinum*, the control efficacy against *Meloidogyne javanica* and *Globodera pallida* can reach above 70% [6]. So far, *P. lilacinum* has been registered as a biocontrol agent to control nematodes [7].

Genomes of four *P. lilacinum* strains have been sequenced [8–10]. According to the transcriptome analysis of *P. lilacinum*, a series of genes annotated as cyclophilins were found to display up-regulated expression when *P. lilacinum* infected nematodes [10]. Cyclophilins (CYPs) are a member of peptidyl prolyl cis-trans isomerases (PPIases), originally characterized as the receptor of an immunosuppressive drug cyclosporine A [11,12]. CYPs possess a highly conserved cyclophilin-like domain (CLD) and are divided into two types. One type is single-domain proteins that contain only the CLD, and the other is multi-domain proteins that harbor functional domains other than CLD, such as WD40, U-box, Leu Zipper, the RNA recognition motif (RRM), and tetratricopeptide repeats (TPRs) [13].

CYPs have been widely identified throughout prokaryotes (e.g., two in *Escherichia coli*) and eukaryote organisms such as fungi, nematodes, plants, animals, and humans [14–23]. CYPs play diverse roles in many cellular processes, including protein folding, cell morphogenesis, cell signaling, transcriptional regulation, RNA splicing, and the response to environmental stress [24–31]. However, the understanding of filamentous fungal CYPs and their potential functions is still preliminary. Studies of individual genes have revealed that CYPs are associated with virulence in filamentous fungi. The gene *BbCypB* with a signal peptide in the insect pathogenic fungus *Beauveria bassiana* contributes positively to virulence during the infection stage [32]. The hCYPA homologs in two plant pathogenic fungi, *Magnaporthe grisea* and *Botrytis cinerea*, were found to be closely related to pathogenicity [33,34].

The genome of *P. lilacinum* strain 36-1 has been sequenced [10]. In this study, we performed a genome-wide analysis of the CYP family members in *P. lilacinum* strain 36-1 (termed PlCYPs), and further explored the phylogenetic relationship of PlCYPs with other CYPs of Ascomycota fungi. We identified candidate *PlCYPs* associated with parasitism to nematodes, and also investigated the function of *PlCYPs* in response to abiotic stresses.

2. Results

2.1. Ten CYP Genes were Identified in P. lilacinum

To identify *CYP* genes in *P. lilacinum*, the amino acid sequences of hCYPA (GenBank: AAI37059.1) were used to search against the *P. lilacinum* strain 36-1 genome and transcriptome database to obtain homologous sequences which were further confirmed by domain analysis [10]. In total, 10 non-redundant *CYP* genes were obtained and named *PlCYP1*–*P10* (Table 1). To confirm the authenticity of acquired genes, the genomic DNA and cDNA of *P. lilacinum* 36-1 were used as templates, and they successfully amplified all of the genes (Figure 1A). The size of these 10 PlCYPs ranged from 162 to 627 amino acids, and the values of isoelectric points varied from 5.81 to 9.49 (Table 1). The prediction of subcellular localization revealed that PlCYP1, 2, 4, 6, 8, and 10 were localized in the cytoplasm. PlCYP3 and PlCYP9 targeted the endoplasmic reticulum and mitochondria, while both PlCYP5 and PlCYP7 were shown to have nuclear localization signals, and were predicted to localize in the nucleus (Table 1). AtCYP59 and SpRct1 are orthologs of PlCYP5 which localized in the nucleus and associated with transcriptional regulation [35,36]. Therefore, the localization of PlCYP5 and PlCYP7 was determined by the transient expression eGFP-tagged fusion proteins in *Nicotiana benthamiana*, suggesting that PlCYP5 and PlCYP7 are localized in the nucleus (Figure 2).

**Table 1.** Characteristics of the *Cyclophilin* (*CYP*) gene family in *Purpureocillium lilacinum* strain 36-1.

| **Gene Name** | **Orthologs in Strain PLFJ-1** | **ORF** (**bp**) | **Deduced Polypeptide** | | | **Predicted Subcellular Localization** |
| --- | --- | --- | --- | --- | --- | --- |
|  |  |  | **Amino Acid** (**aa**) | **Molecular Weight** (**kDa**) | **Isoelectric Point** |  |
| *PlCYP1* | XP_018182827.1 | 489 | 162 | 17.4 | 6.06 | Cytoplasm |
| *PlCYP2* | XP_018178542.1 | 1764 | 587 | 64.0 | 8.49 | Cytoplasm |
| *PlCYP3* | XP_018176208.1 | 627 | 208 | 22.8 | 7.89 | Endoplasmic reticulum |
| *PlCYP4* | XP_018179002.1 | 1119 | 372 | 40.3 | 5.81 | Cytoplasm |
| *PlCYP5* | XP_018178952.1 | 1437 | 478 | 54.8 | 5.93 | Nucleus |
| *PlCYP6* | XP_018179645.1 | 1884 | 627 | 70.2 | 6.49 | Cytoplasm |
| *PlCYP7* | XP_018177409.1 | 1623 | 540 | 60.2 | 5.98 | Nucleus |
| *PlCYP8* | XP_018177405.1 | 507 | 168 | 18.1 | 7.01 | Cytoplasm |
| *PlCYP9* | XP_018174420.1 | 684 | 227 | 24.6 | 9.49 | Mitochondria |
| *PlCYP10* | XP_018175923.1 | 549 | 182 | 19.8 | 6.29 | Cytoplasm |


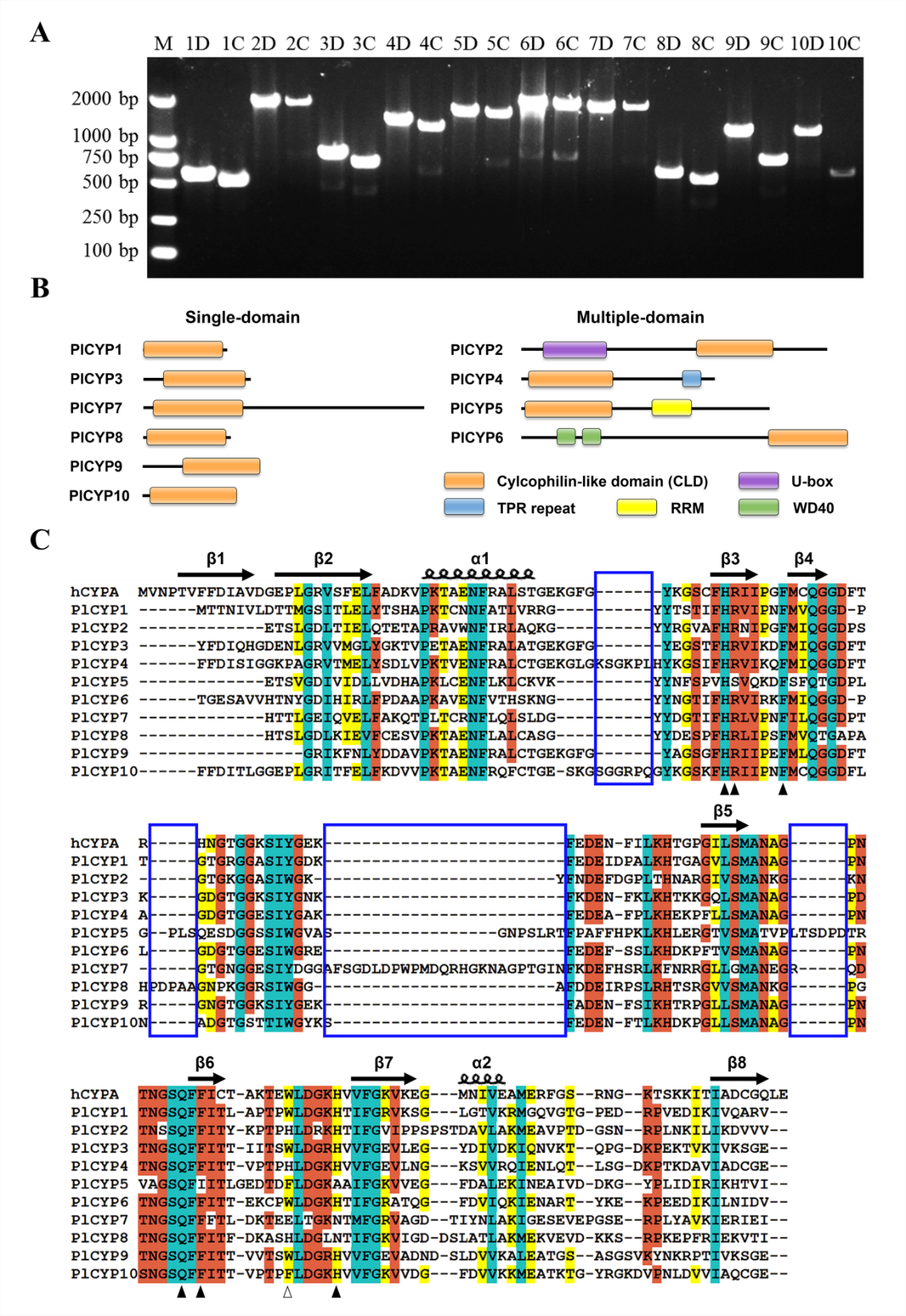


**Figure 1.** Members of the cyclophilin family in *P. lilacinum* (PlCYPs). (**A**) PCR amplification to detect *PlCYP* genes. Lane M is the DNA molecular weight marker, the numbers “1–10” in lanes 2–21 indicate genes *PlCYP1–P10*. The letter “D” indicates DNA as a template, and the letter “C” indicates cDNA as a template. “1D” indicates the use of the genomic DNA of *P. lilacinum* as a template to amplify the *PlCYP1* gene. (**B**) Domains of PlCYPs are shown in proportion to the length of sequences. The single-domain PlCYPs are shown on the left side and the multi-domain PlCYPs are shown on the right side. (**C**) Multiple alignment of the conserved *P. lilacinum* cyclophilin-like domain (CLD) with the human CYPA (hCYPA). The secondary structures are displayed above the sequences. β indicates the β fold, and α indicates the α helix. Residues are shaded by different colors according to the conserved percent: cyan indicates 100%; reddish orange means 80%; and yellow indicates 60%. Solid and hollow triangles denote residues predicted to have peptidyl prolyl cis-trans isomerases (PPIase) activity and a cyclosporin A binding site. The blue boxes represent the gaps between PlCYPs and hCYPA.


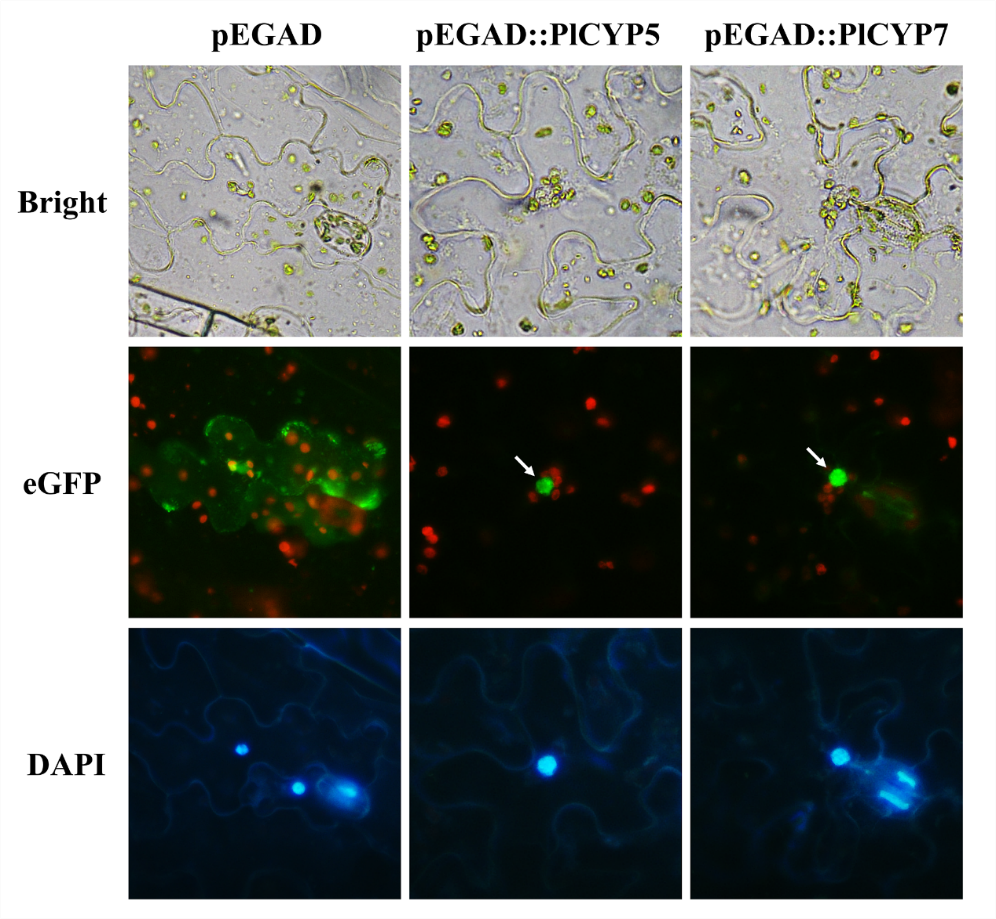


**Figure 2.** Subcellular localizations of PlCYP5 and PlCYP7 in *Nicotiana benthamiana*. *Agrobacterium tumefaciens* was used to transiently express eGFP::PlCYP5 or eGFP::PlCYP7 fusion proteins in the leaves of *N. benthamiana*, and transient expression of eGFP was used as the control. Subcellular localizations of the fused proteins were visualized using fluorescence microscopy at 48 h after infiltration, and 4’, 6-diamidino-2-phenylindole was used to stain the nucleus of epidermal cells before microscopic observation. The arrows indicate proteins localized in the nucleus.

2.2. The PlCYPs Contain the CLD Domain with Residue Variation

The ScanProsite analysis showed that, among the 10 PlCYPs, PlCYP1, 3, 7, 8, 9 and 10 are single-domain proteins that only contain the CLD, while PlCYP2, 4, 5, and 6 possess additional domains. PlCYP2 and PlCYP6 contain a U-box and WD40 domain in the N-terminal of their sequences, while PlCYP4 has a TPR domain in the C-terminal and PlCYP5 possesses an RRM (Figure 1B).

In CYPs, CLD is the PPIase functional domain that contains many conserved residues, including H54, R55, F60, Q111, F113, W121, and H126 specific to hCYPA [37]. To obtain more sequence details, alignment of the CLD region was performed and the hCYPA, which represents the typical CYP, was used as the reference. The result indicated that sequences encoding the secondary structures of CLD in each PlCYP were conserved (Figure 1C). However, the residues for PPIase activity and cyclosporin A binding were partially mutated in PlCYPs such as PlCYP5 and PlCYP7 (Figure 1C, triangle). In addition, PlCYP4, 5, 7, 8, and 10 contained extra short sequences that hCYPA lacks, for which four gaps were shown by multiple alignment analysis (Figure 1C, blue box).

2.3. Phylogenetic Analysis Showed Evolutionary Divergence of CYPs between Ascomycotina and Saccharomycotina Fungi

To explore the evolutionary relationship within fungal CYPs, besides that of CYPs in *P. lilacinum*, sequences of 94 CYPs from different fungal species belonging to the Ascomycota phylum (Ascomycotina and Saccharomycotina) were collected, and used to construct a phylogenetic tree. As observed in Figure S1, the phylogenetic tree was divided into 10 groups (group A to J) based on sequence homology, and the PlCYPs were distributed in different groups. Eight pairs of CYPs from *B. bassiana* and *P. lilacinum* were clustered together, including PlCYP2/BbCYP8, PlCYP3/BbCYPB, PlCYP4/BbCYPD, PlCYP6/BbCYPE, PlCYP7/BbCYP9, PlCYP8/BbCYP3, PlCYP9/BbCYPA, and PlCYP10/BbCYPH. In addition, only groups G, H, and I had CYPs of Saccharomycotina fungi, but their CYPs were separated into the Ascomycotina fungal CYPs in these groups, which formed two independent branches (groups G1/G2, H1/H2 and I1/I2).

To further understand the clustering manner, all labels were tinted with different background colors, representing different predicted cellular localizations. It showed that the CYPs of the same predicted cellular localization tend to cluster together. Proteins predicted to target the mitochondria were distributed in group H, and groups A and B only contained CYPs that were predicted to localize in the nucleus (Figure S1). Moreover, the multi-domain CYPs with similar domain architectures were also clustered together, including groups B, E, F, and I (Figure S1).

2.4. The CYPs in Ascomycotina Fungi Display a More Complex Motif and Gene Structure Distribution than the CYPs in Saccharomycotina

The MEME program was employed to analyze the conserved and potential motifs of all selected fungal CYPs. Twenty motifs were identified, and their distribution was displayed corresponding to the phylogenetic tree (Figure S2A). This showed that the CYPs in the same group have similar motifs (Figure S2B). The sequences of motifs were then annotated (Table S1), showing that six motifs (motif 1, 2, 3, 4, 5, and 7) are part of the CLD and existed in all groups except for group B, which lacks motifs 2 and 5 (Figure S2B). Moreover, some motifs exclusively appeared in a certain group of proteins. For example, only members of group B possess motif 6 (Figure S2B), motifs 9, 10, 12, 16, and 20 are only presented in group F, and motifs 14 and 15 only exist in group I (Figure S2B).

The gene structure analysis showed that intron numbers in coding sequences of the 10 *PlCYPs* vary from 0 to 5. Similar to the motifs, the exon-intron distribution was diverse in different groups (Figure S2C). Additionally, the intron numbers are different for members in the same group. Proteins in group B had intron numbers that range from 0 to 3, whereas, groups G, H, and I which contain both Ascomycotina and Saccharomycotina fungal CYPs exhibited more complex intron numbers and lengths (Figure S2C). Nevertheless, CYPs clustered into pairs display similar distributions, such as PlCYP5/GzCYP8, PlCYP8/BbCYP3, and PlCYP3/BbCYPB.

2.5. The PlCYP6 Gene Has Two Transcripts

Among the 10 *PlCYPs*, *PlCYP6*, whose coding product possesses the CLD and WD40 domain, has the longest length (Figure 1B). The comparison of the transcriptome and genome indicated that *PlCYP6* has two transcripts. To confirm this result, specific primers were used for amplification and sequencing, and two transcripts were obtained, transcript-a (T-a) and transcript-b (T-b) (Figure 3B). Thus, *PlCYP6* has two introns within its coding region, and the second intron can either be spliced or not. In the case of T-a, the second intron is retained, and the translation process will end at the termination codon TAA, which is located in the second intron, otherwise, if the second intron is spliced, the translation process will end at the termination codon TAG (Figure 3A).


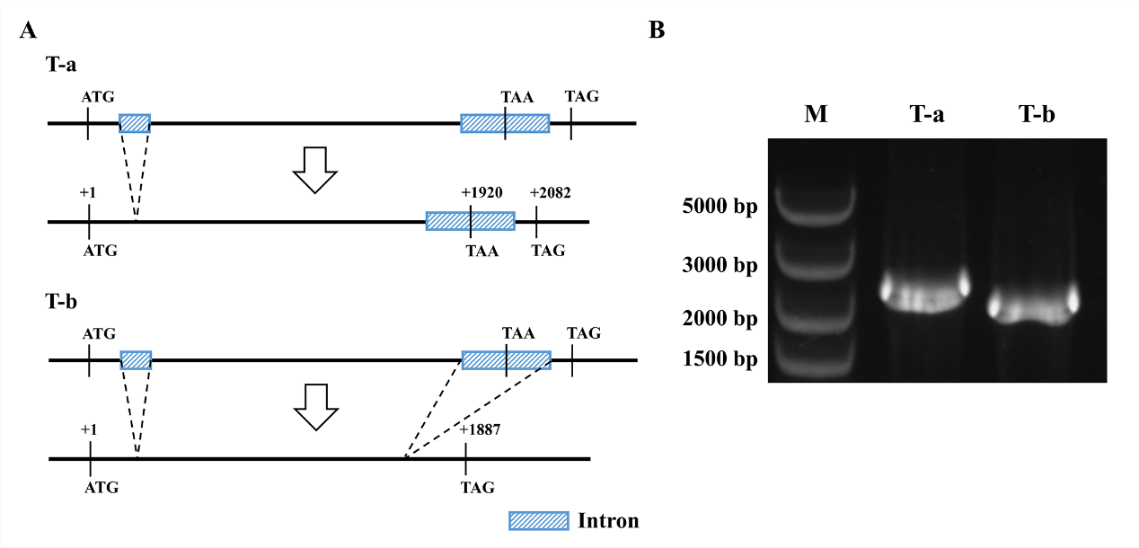


**Figure 3.** Alternative splicing of the *PlCYP6* gene in *P. lilacinum*. (**A**) Diagram of the alternative splicing process, generating two transcripts (T-a and T-b). (**B**) PCR amplification of the two transcripts with specific primers using the cDNA of strain 36-1 as a template.

2.6. PlCYPs Exhibit Different Expression Patterns in Different Fungal Morphologies and at the Egg Parasitic Stage

To overview the roles and activities of PICYPs across fungal developments, their expression levels were assessed using the real-time RT-PCR analyses of total RNAs prepared from conidia, germinating conidia, blastospores, vegetative hyphae, and aerial hyphae of *P. lilacinum* strain 36-1 (Figure 4A). The quantitative results showed that the 10 *PlCYPs* were ubiquitously expressed in all samples (Figure 4B). Compared with un-germinated conidia, all *PlCYPs* except *PlCYP7* were significantly up-regulated after 8 h of germination, and two genes, *PlCYP5* and *PlCYP6*, had significantly higher expressions in blastospores than in conidia (Figure 4B). In addition, compared with the hyphae in the vegetative growth stage, the expression levels of *PlCYPs* in aerial hyphae were almost the same, with only the expressions of *PlCYP4* and *PlCYP9* being significantly decreased (Figure 4B).


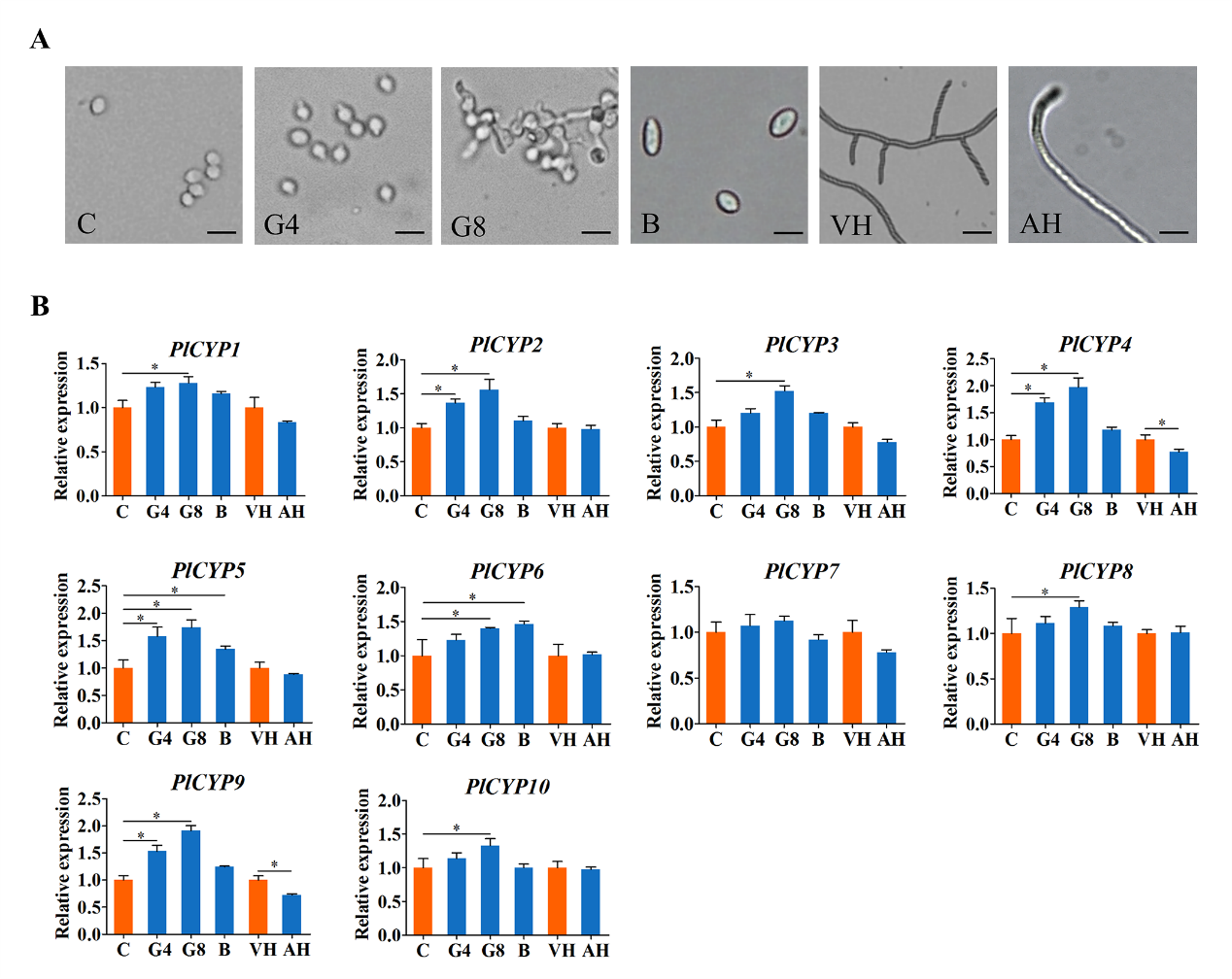


**Figure 4.** Expression levels of *PlCYPs* in different fungal morphologies. (**A**) Microscopic images of *P. lilacinum* at different growth stages. “C” indicates conidia, “G4” indicates conidia germinated for 4 h, “G8” indicates conidia germinated for 8 h, “B” indicates blastospores, “VH” indicates vegetative hyphae, and “AH” indicates aerial hyphae. Scale = 5 μm. (**B**) Relative expression levels of *PlCYPs* in conidia germination for 4 h, germination for 8 h and blastospores versus conidia, and relative expression levels in aerial hyphae versus vegetative hyphae. The β-actin and tubulin genes were used as internal controls to normalize the data. The error bar represents the standard deviation (SD) of three replicates. * denotes *p* < 0.01 in the variance analysis.


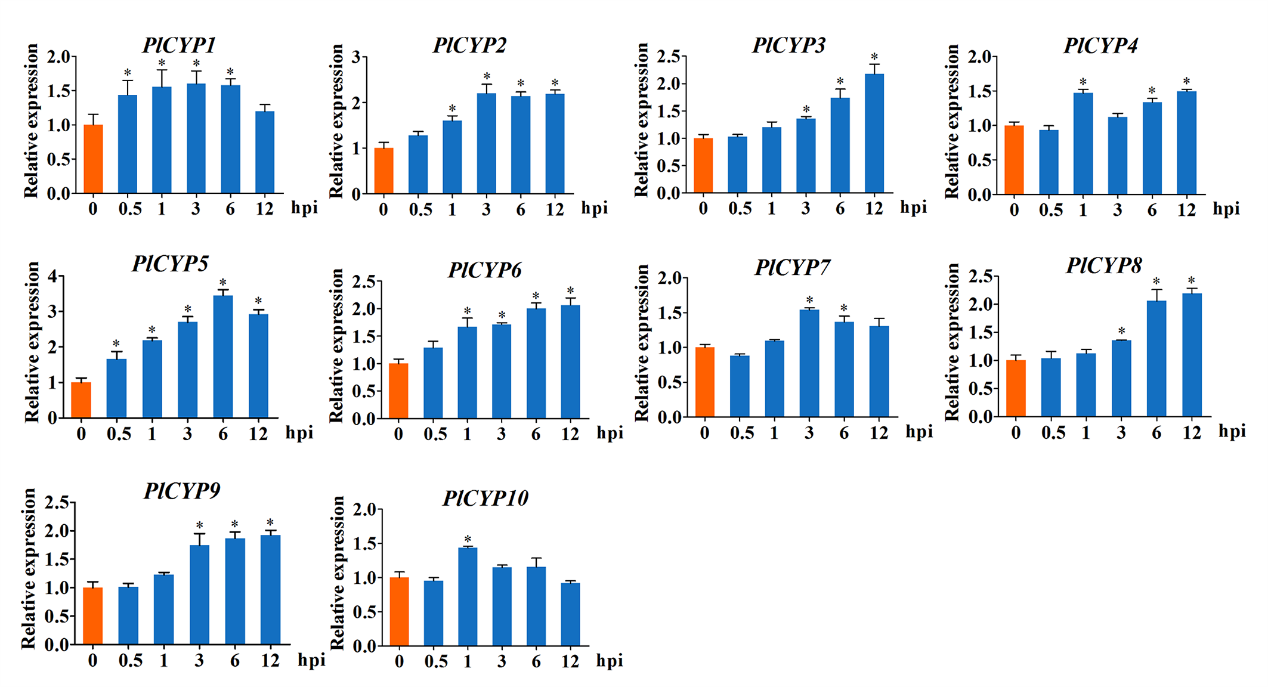


**Figure 5.** Expression levels of *PlCYPs* at the *Meloidogyne incongnita* parasitic egg stage. Relative expression levels of *PlCYPs* at 0.5, 1, 3, 6, and 12 h post inoculation (hpi) versus the control with no eggs. The β-actin and tubulin genes were used as internal controls to normalize the data. The error bar represents the SD of three replicates. * denotes *p* < 0.01 in the variance analysis.

Considering that *P. lilacinum* is an egg-parasite fungus, the eggs of the nematode were inoculated with fungal conidia of *P. lilacinum*, and relative expressions of *PlCYPs* were investigated at different times after inoculation (hpi), with 0 hpi being used as the control. The results showed that the expressions of all 10 *PlCYPs* increased after the inoculation of eggs. Many genes, such as *PlCYP3*, *6*, *8*, and *9*, exhibited significant expression at later stages of inoculation (Figure 5). In particular, *PlCYP5* was significantly up-regulated at all time points with the highest expression at 6 hpi (Figure 5).

2.7. The Expression of PlCYP4 and PlCYP6 were Induced under High Temperature and H_2_O_2_ Stresses

The mycelia were grown on solid medium for long-term stress and stimulated in liquid media for short-term stress. The expression levels of *PlCYPs* were analyzed by comparing similarities and differences between long-term and short-term stress response tests (Figure S3). As shown in Figure 6, there are differences between the two results. All *PlCYPs* were down-regulated when *P. lilacinum* grew on the plates containing 1 M NaCl and 1.2 M sorbitol, whereas *PlCYP2, 5,* and *6* were positively induced during the short-term response period (Figure 6). Nevertheless, two genes showed consistency in their expression levels. *PlCYP6* showed the most active response to H_2_O_2_ both in the long-term and short-term assays, and the *PlCYP4* maintained the highest expression in both assays after high temperature treatment (Figure 6).


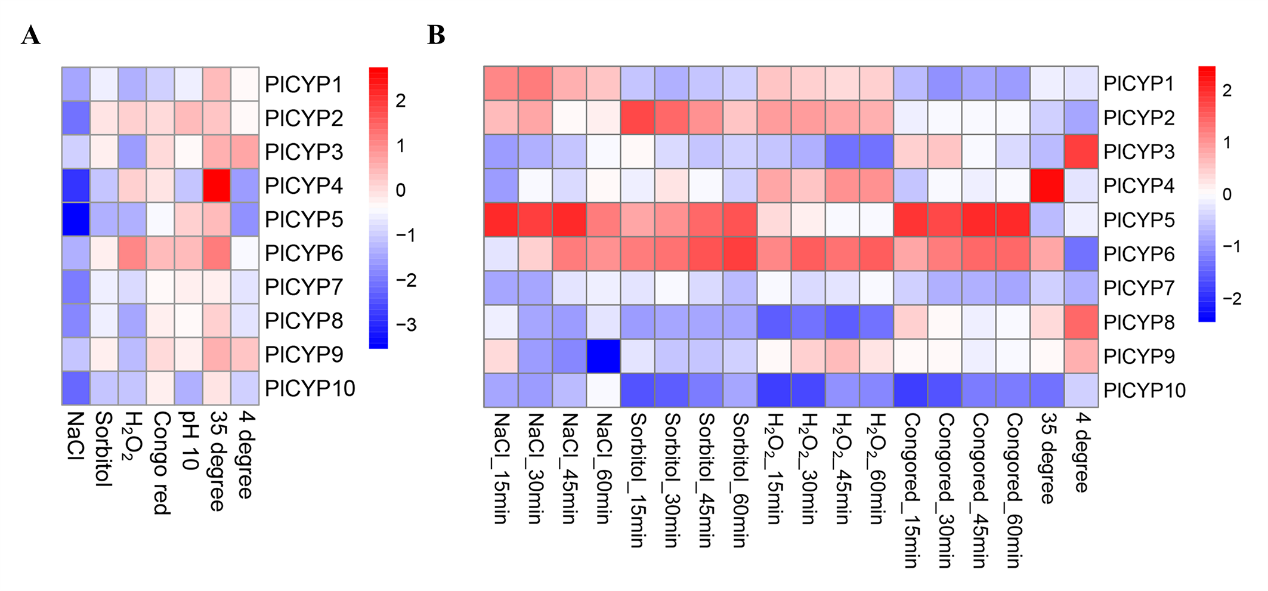


**Figure 6.** Expression of *PlCYPs* under different abiotic stresses. (**A**) Relative expression of *PlCYPs* on solid Czapek-Dox medium (CZM) media under different abiotic stresses, including 1 M NaCl, 1.2 M sorbitol, 5 mM H_2_O_2_, 0.15 mg/mL Congo red, alkaline stress (pH 10), and temperature stress (35 and 4 °C). (**B**) Relative expressions of *PlCYPs* in liquid CZM media under the above-mentioned abiotic stresses. The fold changes of gene expressions were calculated versus the control by the 2^−△△Ct^ method, and the logarithm (log 2) of those values was applied to generate the heat map.

2.8. Heterologous Expressions of PlCYP4 and PlCYP6 in E. coli Enhance Tolerance towards Abiotic Stresses

The expression results suggest that *PlCYP4* and *PlCYP6* have higher expressions in response to high temperature and the presence of H_2_O_2_, respectively (Figure 6). Therefore, heterologous expressions of *PlCYP*s in *E. coli* were determined based on the growth of *E. coli* on Luria-Bertani (LB) solid media supplemented with different abiotic stresses to detect whether *PlCYP4* and *PlCYP6* are involved in abiotic stress tolerance (Figure S4). Due to *PlCYP6* having alternative splicing, heterologous expressions of transcripts T-a and T-b were both tested. The *E. coli* cultured on LB basal plates was used as the control, showing that the growth of recombinants and the wild type strain was similar (Figure S5). On the LB plate supplemented with 1 mM H_2_O_2_, both transcripts T-a and T-b of the *PlCYP6* recombinants grew, but the control and *PlCYP4* recombinant were unable to grow (Figure S5). On LB plates containing 600 mM NaCl, 800 mM sorbitol, or LB medium for which the pH was adjusted to 10, tolerance was not displayed by any of the recombinants (Figure S5).

In order to quantify the growth rate, the liquid culture method was adopted under the conditions of H_2_O_2_ and a high temperature. The liquid cultures were diluted to appropriate concentrations and spread on LB media to count the colony numbers. As expected, in liquid culture containing H_2_O_2_, the growth of *E. coli* recombinants expressed T-a and T-b better than in the wild type control (Figure 7A). In addition, after being treated at 70 °C for 20 min, the colonies of the *PlCYP4* recombinant were significantly more numerous than with the control (Figure 7B).


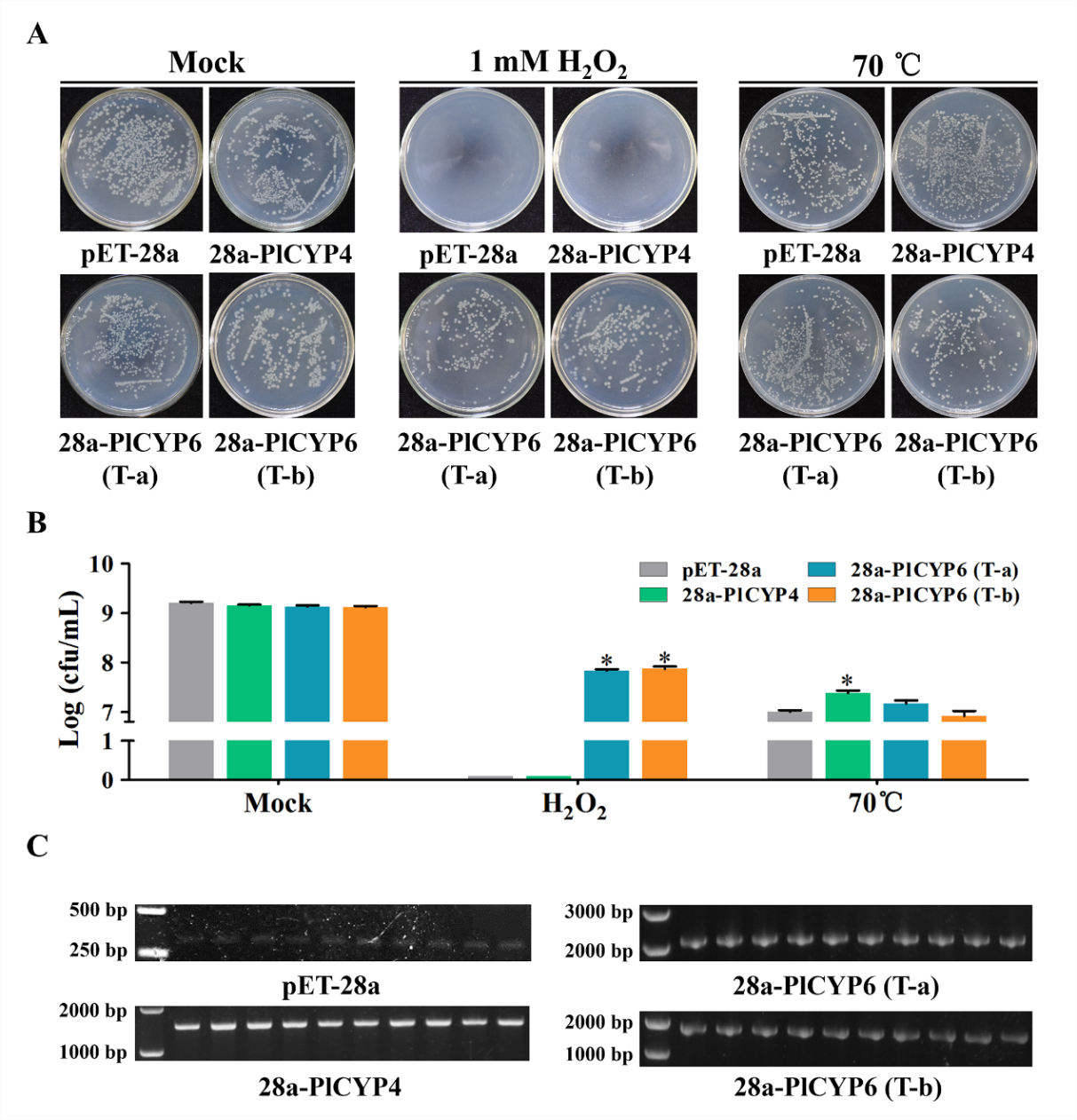


**Figure 7.** Assay of *PlCYPs*’ response to different abiotic stresses. (**A**) Growth analysis of *E. coli* cells containing an empty vector (pET-28a) and recombinant vectors (pET-PlCYP4 and pET-PlCYP6) was carried on Luria-Bertani (LB) liquid medium with two treatments (supplemented with 1 mM H_2_O_2_ and 70 °C for 20 min). The treated cultures were diluted to a suitable concentration and then spread onto the LB plate to count the number of living bacteria. (**B**) Statistical analysis of the bacterial colonies. The error bar represents the SD of three replicates. * denotes *p* < 0.01.

3. Discussion

*P. lilacinum* has a high efficiency in terms of parasitizing nematode eggs and is beneficial for plant growth, making it an effective biocontrol agent for controlling plant parasitic nematodes. However, there are obstacles in the large-scale application of this fungal agent, such as the difficulty in maintaining its activity and its adaption to unfavorable environments, which lead to a short shelf life and unstable field biocontrol effect. Understanding the molecular mechanisms of nematode parasitism and adaption to adversity will promote the application of this fungus in plant protection. The current study revealed the composition of members of the *Cyclophilin* gene family in *P. lilacinum* by profiling their expression patterns during development and nematode egg infection and analyzing their potential functions. These results provide a framework for future studies and the use of CYPs in *P. lilacinum*.

In the present study, 10 *PlCYPs* were identified through a genome-wide analysis. Within the fungi analyzed in this study, the Ascomycotina fungi were shown to have approximately 10 CYP proteins, whereas Saccharomycotina such as *C. albicans*, *C. glabrata,* and *S. cerevisiae* have 6, 6, and 8 CYPs, respectively, supporting that Saccharomycotina has a lower number of CYPs than Ascomycotina [38]. In addition, previous research showed that prokaryotes generally have less CYPs than eukaryotes [14].

Phylogenetic analysis of 10 PlCYPs in *P. lilacinum* with 84 CYPs identified in nine Ascomycota fungi, and generated a tree that was artificially divided into 10 groups. The phylogenetic tree did not show a close relationship within the 10 PlCYPs, which were distributed into separated groups with no paralog. This is probably associated with the functional diversity of the PlCYPs. In addition, three groups G, H, and I were found to possess all of the analyzed species, while the CYPs of Ascomycotina and Saccharomycotina fungi were clearly separated into two branches, suggesting evolutionary divergence between the two kinds of fungi. Motif and gene structure analysis is an effective tool that can be employed to understand sequence signatures in plant research [39-41]. The motif distributions and gene structures of fungal CYPs associated with the phylogenetic tree were exhibited. We found that fungal CYPs in the same group had similar motif arrangements, and six motifs (motifs 1–5 and 7) were annotated as having the conserved CLD, similar to the CYPs in plants. In particular, members in group B, such as PlCYP5 were found to lack motifs 2 and 5, suggesting that proteins in this group may possess a degenerated CLD with a weak catalytic capacity. Overall, the motifs and gene structure analyses provided additional evidence to support the phylogenetic relationship.

The cyclophilin-like domains in the PlCYPs sequences were highly conserved, which is consistent with the previous description [42]. However, the PlCYPs varied widely in terms of sequence length, isoelectric point, and subcellular localization, providing another piece of evidence for functional diversity of PlCYPs. In the PPIase superfamily, the CYP family is the only one that has members which localize in the mitochondria [42]. However, the subcellular localization of PlCYPs did not show a close relationship with the phylogenetic analysis. For instance, both PlCYP5 and PlCYP7 were predicted to localize in the nucleus, but PlCYP5 has an RRM domain and was classified into group B, and PlCYP7 which does not harbor an RRM domain and was placed into group A. This indicates that PlCYPs in the same cellular place play different roles, supporting a previous observation that it is insufficient to regard localization as the sole criteria for classification of CYPs [21].

The expression profiles of *PlCYPs* in different fungal morphologies, during the process of egg parasitism and under abiotic stresses, indicated that the 10 PlCYPs appear to have a wide range of diverse roles within the cell. Previous studies have demonstrated that plant CYPs have tissue-specific expression [23]. In our results, PlCYPs showed a ubiquitous expression abundance at different stages of fungal development, which is different from plant CYPs. In addition, we measured the expressions of *PlCYPs* at different time points after egg inoculation, and the results confirmed the transcriptome data (Figure S6), demonstrating that the expressions of all *PlCYPs* were up-regulated to varying degrees. *PlCYP5* was significantly expressed at 0.5 hpi, suggesting PlCYP5 might have a similar function as its orthologs AtCYP59 and SpRct1 (Table S2) that are involved in transcriptional regulation [35,36]. The expression of *PlCYP3* was increased significantly after 3 hpi, suggesting that *PlCYP3* may assist in the eggshell invasion of hyphae. In addition, other genes, such as *PlCYP2*, *6*, *8*, and *9*, showed significant expression levels at later stages of inoculation, indicating their potential roles in egg parasitism.

It has been shown that CYP proteins play crucial roles in abiotic stress tolerance [22,23]. For *PlCYPs*, the long- and short-term responses to abiotic stresses were measured. There were differences between the two results, suggesting different mechanisms of response. The expression of *PlCYP5* increased during short-term responses to stresses but decreased in long-term responses, implying that it may be involved in early-stage regulation of genes related to the stress response. Nevertheless, *PlCYP4* exhibited a higher expression level in both the short- and long-term responses to high temperature. Similarly, *PlCYP6* had a higher expression level in both short- and long-term response to H_2_O_2_. This indicates that *PlCYP4* and *PlCYP6* may be related to high temperature and H_2_O_2_ stress tolerance. In addition, the related functions of PlCYP4 and PlCYP6 were further proved by the heterologous expression assay. This study indicates the potential functions of PlCYPs in pathogenicity and the abiotic stress responses, and also provides a frame for further analysis of the *CYP* gene family in Ascomycotina fungi. However, the accurate functions of PlCYPs in *P. lilacinum* need to be explored in the future.

4. Materials and Methods

4.1. Fungal Strains and Growth Condition

The fungus *P. lilacinum* strain 36-1 was isolated from the egg surface of *Meloidogyne incognita* from field soil from Hubei Province in China. Strain 36-1 was cultured on a potato dextrose agar (PDA) plate at 28 °C for normal culture and on Czapek-Dox medium (CZM) at 28 °C for phenotypic determination. It was stored at 4 °C.

4.2. Identification and Classification of CYP Genes in P. lilacinum Strain 36-1

To identify *CYP* genes in the genome of *P. lilacinum* strain 36-1, a local BLASTP search was performed using the amino acid sequences of hCYPA (GenBank: AAI37059.1). The generated sequences were searched again for other PlCYPs until no new sequence appeared. ScanProsite (<https://prosite.expasy.org/>) and the Conserved Domain Database (CDD) were used to ensure that the candidate PlCYPs harbored a cyclophilin-like domain (CLD). The genome of the *P. lilacinum* strain PLFJ-1 (Accession: LSBI00000000) was regarded as another database for validation, using the same searching procedure.

The coding sequences of PlCYPs were obtained by comparing the genomic and transcriptome databases [10]. The molecular weight (kD) and isoelectric point (pI) of each protein were predicted by Expasy programs using amino acid sequences (<http://www.expasy.org/tools/>). The WoLF PSORT II program (<https://www.genscript.com/wolf-psort.html>) was used for the prediction of subcellular localizations of PlCYPs.

4.3. Protein Alignment and Phylogenetic Analysis

The full-length coding sequences of PlCYPs were aligned by the ClusterW program with default parameters. To show the residual variation of CLD, sequences of the CLD were singled out for alignment in the same manner. For phylogenetic analysis, amino acid sequences of the fungal CYPs were downloaded from National Center for Biotechnology Information (NCBI, Rockville Pike, Bethesda MD, USA) by accession number (Table S3), and the sequences were aligned using the MUSCLE program of MEGA 6.0 with default settings. MEGA 6.0 software (<http://www.megasoftware.net/>) was employed to construct the phylogenetic tree using the neighbor-joining (NJ) method with 1000 bootstrap replicates. The tree was exported into the Interactive Tree of Life (<http://itol.embl.de>) for further annotation.

4.4. Motifs and Gene Structure Analyses

The MEME (<http://meme-suite.org/tools/meme>) was used to analyze conserved and potential motifs with the parameter settings: any number of repetitions, a minimum motif width of 6, a maximum motif width of 200, and a maximum number of motifs of 20. Subsequently, the created motifs were annotated by the Pfam (<http://pfam.xfam.org/>). Exon-intron organization was predicted using Gene Structure Display Server 2.0 (GSDS 2.0) (<http://gsds.cbi.pku.edu.cn/>) by comparing the coding sequences with corresponding DNA sequences.

4.5. Transient Expression of Tobacco

The open reading frames of *PlCYP5* and *PlCYP7* were cloned into the vector pEGAD to generate the vectors pEGAD::PlCYP5 and PEGAD::PlCYP7 [43]. The recombinant vectors were transferred into *Agrobacterium tumefaciens* GV3101 by electroporation. The cultured *A. tumefaciens* cells (OD_600_ = 0.4) were infiltrated into the leaves of *Nicotiana benthamiana* which were grown in the greenhouse for four weeks at 26 °C under a 16 h light/8 h dark cycle. The same process using the empty vector pEGAD was used as a control. Subcellular localizations of the fused proteins were visualized using fluorescence microscopy at 48 h after infiltration.

4.6. Sample Treatments for qRT-PCR

Different fungal morphological samples were collected from *P. lilacinum* strain 36-1, including conidia, germinating conidia, blastospores, vegetative mycelia, and aerial mycelia. Briefly, the conidia were washed from *P. lilacinum* strain 36-1 that had been cultured on PDA for three weeks. For germination, the conidia were standardized to 1 × 10^7^ conidia per milliliter and added to liquid CZM with a dilution ratio of 1:100 for shaking culture, and the samples were then collected at 4 and 8 h post inoculation (hpi). To produce blastospores, we conducted the study based on previously reported methods for *B. bassiana* with modifications; namely, potato dextrose broth (PDB) was used instead of Sabouraud’s medium [44]. Vegetative and aerial mycelia were separated and collected from the fungus that was cultured for one week on PDA. All collected samples were immediately used for RNA extraction.

Mycelia-infecting nematode eggs were collected by a previously described method [10]. Specifically, *M. incongnita* egg-masses were isolated from tomato roots and suspended in sterile distilled water after being sterilized with 0.5% (*v*/*v*) NaClO. The conidia of *P. lilacinum* strain 36-1 were pre-cultured in liquid minimal media for 24 h and washed by sterile water. Then, the pre-cultured conidia were mixed with *M. incongnita* eggs. Mycelia were collected at 0, 0.5, 1, 3, 6, and 12 hpi by filtering.

In the abiotic stress treatment assay, the conidia were standardized to 1 × 10^5^ conidia per mL, and 50 μL of conidia was spread on CZM supplemented with 1 M NaCl, 1.2 M sorbitol, 5 mM H_2_O_2_, or 0.15 mg/mL Congo red, and the pH was adjusted to 10. Strain 36-1 cultured on normal CZM was regarded as the control. The mycelia of the strain 36-1 were collected at 3 dpi. To test the expressions of *PlCYPs* under different temperatures, the *P. lilacinum* 36-1 strains were cultured on normal CZM for three days, and were then were cultured at 4 or 35 °C for 1 h.

A liquid assay was also performed to detect the short-term expressions of *PlCYPs* under the same abiotic stress conditions. The conidia were inoculated in 150 mL liquid CZM and shock culture for two days. After filtration, the collected mycelia were immediately transferred to the fresh liquid CZM containing the same abiotic stresses for further shaking culture. The mycelia were collected at 15, 30, 45, and 60 min. All of the above-mentioned assays were performed with three biological replicates.

4.7. RNA Extraction and qRT-PCR

Total RNA was isolated with TRIzol reagent (Invitrogen^TM^, Carlsbad, CA, USA) according to the manufacturer’s protocols. The total RNA was treated with the DNA-free™ DNA Removal Kit (Invitrogen^TM^, Carlsbad, CA, USA), and was then used to generate the first strand of cDNA with the RevertAid First Strand cDNA Synthesis Kit (Thermo Scientific, Rockford, IL, USA). Primers used for qRT-PCR are listed in Table S4. The amplification efficiencies were individually verified and are shown in Figure S7. Gene expression abundance was analyzed by qRT-PCR using the Bio-Rad CFX96 Real Time System and SsoFast^TM^ EvaGreen Supermix (Bio-Rad, Hercules, CA, USA). The fold changes of gene expression were calculated versus the control by the 2^−△△Ct^ method (Table S5), which was applied to draw a heat map using the pheatmap function in R. β-actin and tubulin were used as endogenous reference genes [9,10].

4.8. Phenotypic Assay of PlCYP4 and PlCYP6 in E. coli

Heterologous expressions of *PlCYP4* and *PlCYP6* in *E. coli* were carried out to detect whether these genes enhance the tolerance of *E. coli* to abiotic stress. The pET-28a(+) plasmid was used as the original vector and the full-length CDS fragments of selected *PlCYP* genes were inserted between restriction sites *Bam*H I and *Eco*R I to generate recombinant expression vectors. Rosetta (DE3) cells containing the recombinant vectors or an empty vector were grown in 10 mL of Luria-Bertani (LB) medium with 50 μg/mL kanamycin until the OD_600_ was about 0.6. A final concentration of 0.5 mmol/L β-d-thiogalactopyranoside (IPTG) was then added into the medium and *E. coli* cells were continually shaken at 16 °C and 150 rpm for 18 h. Finally, all samples were adjusted to OD_600_ = 1.0 and then further diluted to 10**^−^**^1^, 10**^−^**^2^, and 10**^−^**^3^. In total, 2 μL of each dilution was spotted on LB plates containing 600 mM NaCl, 800 mM sorbitol or 1 mM H_2_O_2_, and LB (pH 10).

Overnight, the transformants were grown overnight in LB liquid medium containing 50 μg/mL kanamycin, and then all samples were diluted to OD_600_ = 0.1. In total, a 10 μL aliquot of solution was added into 10 mL of LB liquid medium that contained 0.5 mM IPTG and 1 mM H_2_O_2_. This was shaken at 37 °C and 180 rpm for 12 h. The strains cultured in LB without H_2_O_2_ were used as controls. In addition, partial cultures of the control group were treated at 70 °C for 20 min. The above-mentioned assays were performed with three replicates.

4.9. Statistical Analysis

Data quantified from the qRT-PCR of three biological replicates were subjected to two-way ANOVA analysis, followed by Bonferroni’s post-test for data comparison. A *p*-value of less than 0.01 was deemed to represent a significant difference.

**Supplementary Materials:**

**Author Contributions:** Y.-N.X. and X.-Q.X. designed this study. C.-M.M. and C.X. carried out the bioinformatics analyses. C.-M.M. and C.X. performed the qRT-PCR experiments. J.L. and Q.-Y.H. helped in the phenotype experiments. C.-M.M. wrote the manuscript. Y.-N.X., X.-Q.X. and G.-F.W. helped in discussions of the manuscript. All authors read and approved the final manuscript.

**Funding:** This research was funded by the national natural science foundation of China, grant number 31872019 and national key R&D program of China, grant number 2018YFD0200500, 2018YFD0201000

**Conflicts of Interest:** The authors declare no conflict of interest.

References

1. Abad, P.; Gouzy, J.; Aury, J.; Castagnone-Sereno, P.; Danchin, E.G.J.; Deleury, E.; Caillaud, M.C. Genome sequence of the metazoan plant-parasitic nematode *Meloidogyne incognita*. *Nat. Biotechnol.* **2008**, *26*, 909–915. doi:10.1038/nbt.1482.
2. Jones, J.T.; Haegeman, A.; Danchin, E.G.J.; Gaur, H.S.; Helder, J.; Jones, M.G.K.; Perry, R.N. Top 10 plant-parasitic nematodes in molecular plant pathology. *Mol. Plant Pathol.* **2013**, *14*, 946–961. doi:10.1111/mpp.12057.
3. Atkins, S.D.; Clark, I.M.; Pande, S.; Hirsch, P.R.; Kerry, B.R. The use of real-time PCR and species-specific primers for the identification and monitoring of *Paecilomyces lilacinus*. *FEMS Microbiol*. *Ecol*. **2005**, *51*, 257–264. doi:10.1016/j.femsec.2004.09.002.
4. Anastasiadis, I.A.; Giannakou, I.O.; Prophetou-Athanasiadou, D.A.; Gowen, S.R. The combined effect of the application of a biocontrol agent *Paecilomyces lilacinus*, with various practices for the control of root-knot nematodes. *Crop Prot*. **2008**, 27, 352–361. doi:10.1016/j.cropro.2007.06.008.
5. Huang, W.K.; Cui, J.K.; Liu, S.M.; Kong, L.A.; Wu, Q.S.; Peng, H.; Peng, D.L. Testing various biocontrol agents against the root-knot nematode (*Meloidogyne incognita*) in cucumber plants identifies a combination of *Syncephalastrum racemosum* and *Paecilomyces lilacinus* as being most effective. *Biol*. *Control* **2016**, *92*, 31–37. doi:10.1016/j.biocontrol.2015.09.008.
6. Hajji, L.; Hlaoua, W.; Regaieg, H.; Horrigue-Raouani, N. Biocontrol potential of *Verticillium leptobactrum* and *Purpureocillium lilacinum* Against *Meloidogyne javanica* and *Globodera pallida* on Potato (*Solanum tuberosum*). *Am*. *J*. *Patato Res*. **2017**, *94*, 178–183. doi:10.1007/s12230-016-9554-0.
7. Kiewnick, S.; Neumann, S.; Sikora, R.A.; Frey, J.E. Effect of *Meloidogyne incognita* inoculum density and application rate of *Paecilomyces lilacinus* strain 251 on biocontrol efficacy and colonization of egg masses analyzed by real-time quantitative PCR. *Phytopathology* **2011**, *101*, 105–112. doi:10.1094/PHYTO-03-10-0090.
8. Prasad, P.; Varshney, D.; Adholeya, A. Whole genome annotation and comparative genomic analyses of bio-control fungus *Purpureocillium lilacinum*. *BMC Genom.* **2015**, *16*, 1004. doi:10.1186/s12864-015-2229-2.
9. Wang, G.; Liu, Z.G.; Lin, R.M.; Li, E.F.; Mao, Z.C.; Ling, J.; Xie, B. Biosynthesis of antibiotic leucinostatins in bio-control fungus *purpureocillium lilacinum* and their inhibition on *Phytophthora* revealed by genome mining. *PloS Pathog*. **2016**, *12*, e1005685. doi:10.1371/journal.ppat.1005685.
10. Xie, J.L.; Li, S.J.; Mo, C.M.; Xiao, X.Q.; Peng, D.L.; Wang, G.F.; Xiao, Y. Genome and transcriptome sequences reveal the specific parasitism of the nematophagous *Purpureocillium lilacinum* 36-1. *Front*. *Microbiol*. **2016**, *7*, 1084. doi:10.3389/fmicb.2016.01084.
11. Handschumacher, R.E.; Harding, M.W.; Rice, J.; Drugge, R.J.; Speicher, D.W. Cyclophilin: A specific cytosolic binding protein for cyclosporin A. *Science* **1984**, *226*, 544–547. doi:10.1126/science.6238408.
12. Gothel, S.F.; Marahiel, M.A. Peptidyl-prolyl cis-trans isomerases, a superfamily of ubiquitous folding catalysts. *Cell*. *Mol*. *Life Sci*. **1999**, *55*, 423–436. doi:10.1007/s000180050299.
13. Schiene-Fischer, C. Multidomain peptidyl prolyl cis/trans isomerases. *Biochim. Biophys. Acta, Gen. Subj.* **2014**, *1850*, 2005–2016. doi:10.1016/j.bbagen.2014.11.012.
14. Hayano, T.; Takahashi, N.; Kato, S.; Maki, N.; Suzuki, M. Two distinct forms of peptidylprolyl-cis-trans-isomerase are expressed separately in periplasmic and cytoplasmic compartments of *Escherichia coli* cells. *Biochemistry* **1991**, *30*, 3041–3048. doi:10.1021/bi00226a009.
15. Andrzej, G. Peptidylprolyl cis/trans isomerases (immunophilins): Biological diversity-targets-functions. *Curr*. *Top*. *Med*. *Chem*. **2003**, *3*, 1315–1347. doi:10.2174/1568026033451862.
16. Liu, J.; Farmer, J.D.; Lane, W.S.; Friedman, J.; Weissman, I.; Schreiber, S.L. Calcineurin is a common target of cyclophilin-cyclosporin A and FKBP-FK506 complexes. *Cell* **1991**, *66*, 807–815. doi:10.1016/0092-8674(91)90124-H.
17. Pemberton, T.J.; Kay, J.E. Identification and comparative analysis of the peptidyl-prolyl cis/trans isomerase repertoires of *H. sapiens*, *D. melanogaster*, *C. elegans*, *S. cerevisiae* and *Sz. pombe*. *Comp*. *Funct*. *Genomics* **2005**, *6*, 277–300. doi:10.1002/cfg.482.
18. Gan, P.H.P.; Shan, W.X.; Blackman, L.M.; Hardham, A.R. Characterization of cyclophilin-encoding genes in *Phytophthora*. *Mol*. *Genet*. *Genom.* **2009**, *281*, 565–578. doi:10.1007/s00438-009-0431-0.
19. Arevalo-Rodriguez, M.; Wu, X.; Hanes, S.D.; Heitman, J. Prolyl isomerases in yeast. *Front. Biosci*. **2004**, *9*, 2420–2446. doi:10.2741/1405.
20. Dolinski, K.; Muir, S.; Cardenas, M.; Heitman, J. All cyclophilins and FK506 binding proteins are, individually and collectively, dispensable for viability in *Saccharomyces cerevisiae*. *PNAS* **1997**, *94*, 13093–13098. doi:10.1073/pnas.94.24.13093.
21. Trivedi, D.K.; Yadav, S.; Vaid, N.; Tuteja, N. Genome wide analysis of Cyclophilin gene family from rice and *Arabidopsis* and its comparison with yeast. *Plant Signal. Behav*. **2012**, *7*, 1653–1666. doi:10.4161/psb.22306.
22. Mainali, H.R.; Chapman, P.; Dhaubhadel, S. Genome-wide analysis of Cyclophilin gene family in soybean (*Glycine max*). *BMC Plant Biol*. **2014**, *14*, 282. doi:10.1186/s12870-014-0282-7.
23. Wang, Q.Q.; Wang, Y.; Chai, W.B.; Song, N.N.; Wang, J.; Cao, L.M.; Li, X. Systematic analysis of the maize cyclophilin gene family reveals ZmCYP15 involved in abiotic stress response. *Plant Cell Tissue Organ Cult.* **2017**, *128*, 543–561. doi:10.1007/s11240-016-1132-0.
24. Anna, K.; Janine, B.; Anne-Marie, K.; Jean, C.; Maria, J.D.; Catherine, K. KIN241: A gene involved in cell morphogenesis in *Paramecium tetraurelia* reveals a novel protein family of cyclophilin-RNA interacting proteins (CRIPs) conserved from fission yeast to man. *Mol*. *Microbiol*. **2001**, *42*, 257–267. doi:10.1046/j.1365-2958.2001.02634.x.
25. Duina, A.A.; Chang, H.C.; Marsh, J.A.; Lindquist, S.; Gaber, R.F. A cyclophilin function in Hsp90-dependent signal transduction. *Science* **1996**, *274*, 1713–1715. doi:10.1126/science.274.5293.1713.
26. Horowitz, D.S.; Lee, E.J.; Mabon, S.A.; Misteli, T. A cyclophilin functions in pre-mRNA splicing. *EMBO* *J*. **2002**, *21*, 470–480. doi:10.1093/emboj/21.3.470.
27. Shaw, P.E. Peptidyl-prolyl isomerases: A new twist to transcription. *EMBO* *Rep*. **2002**, *3*, 521–526. doi:10.1093/embo-reports/kvf118.
28. Kumari, S.; Singh, P.; Singla-Pareek, S.L.; Pareek, A. Heterologous expression of a salinity and developmentally regulated rice cyclophilin gene (*OsCyp2*) in *E. coli* and *S. cerevisiae* confers tolerance towards multiple abiotic stresses. *Mol*. *Biotechnol*. **2009**, *43*, 195–204. doi:10.1007/s12033-009-9153-0.
29. Ruan, S.L.; Ma, H.S.; Wang, S.H.; Fu, Y.P.; Xin, Y.; Liu, W.Z.; Chen, H.Z. Proteomic identifcation of OsCYP2, a rice cyclophilin that confers salt tolerance in rice (Oryza sativa L.) seedlings when overexpressed. *BMC* *Plant Biol.* **2011**, *11*, 1117–1120. doi:10.1186/1471-2229-11-34.
30. Trivedi, D.K.; Ansari, M.W.; Tuteja, N. Multiple abiotic stress responsive rice cyclophilin: (OsCYP-25) mediates a wide range of cellular responses. *Commun. Integr. Biol.* **2013**, *6*, e25260. doi:10.4161/cib.25260.
31. Kim, S.K.; You, Y.N.; Park, J.C.; Joung, Y.; Kim, B.G.; Ahn, J.C.; Cho, H.S. The rice thylakoid luminal cyclophilin OsCYP20-2 confers enhanced environmental stress tolerance in tobacco and Arabidopsis. *Plant Cell Rep.* **2012**, *31*, 417–426. doi:10.1007/s00299-011-1176-x.
32. Chu, Z.J.; Sun, H.H.; Ying, S.H.; Feng, M.G. Vital role for cyclophilin B (CypB) in asexual development, dimorphic transition and virulence of *Beauveria bassiana*. *Fungal Genet*. *Biol*. **2017**, *105*, 8–15. doi:10.1016/j.fgb.2017.05.004.
33. Viaud, M.C.; Balhadere, P.V.; Talbot, N.J. A *Magnaporthe grisea* cyclophilin acts as a virulence determinant during plant infection. *Plant Cell* **2002**, *14*, 917–930. doi:10.1105/tpc.010389.
34. Viaud, M.; Brunet-Simon, A.; Brygoo, Y.; Pradier, J.M.; Levis, C. Cyclophilin A and calcineurin functions investigated by gene inactivation, cyclosporin A inhibition and cDNA arrays approaches in the phytopathogenic fungus *Botrytis cinerea*. *Mol*. *Microbiol*. **2003**, *50*, 1451–1465. doi:10.1046/j.1365-2958.2003.03798.x. Gullerova, M.; Barta, A.; Lorkovic, Z.J.
35. AtCyp59 is a multidomain cyclophilin from *Arabidopsis thaliana* that interacts with SR proteins and the C-terminal domain of the RNA polymerase II. *RNA* **2006**, *12*, 631–643. doi:10.1261/rna.2226106.
36. Gullerova, M.; Barta, A.; Lorkovic, Z.J. Rct1, a nuclear RNA recognition motif-containing cyclophilin, regulates phosphorylation of the RNA polymerase II C-terminal domain. *Mol*. *Cell*. *Biol*. **2007**, *27*, 3601–3611. doi:10.1128/MCB.02187-06.
37. Zydowsky, L.D.; Etzkorn, F.A.; Chang, H.Y.; Ferguson, S.B.; Stolz, L.A.; Ho, S.; Walsh, C.T. Active-site mutants of human cyclophilin-a separate peptidyl-prolyl isomerase activity from cyclosporine-a binding and calcineurin inhibition. *Protein Sci.* **1992**, *1*, 1092–1099. doi:10.1002/pro.5560010903.
38. Pemberton, T.J. Identification and comparative analysis of sixteen fungal peptidyl-prolyl cis/trans isomerase repertoires. *BMC Genom.* **2006**, *7*, 244. doi:10.1186/1471-2164-7-244.
39. Han, Y.H.; Ding, T.; Su, B.; Jiang, H.Y. Genome-wide identification, characterization and expression analysis of the chalcone synthase family in maize. *Int. J. Mol. Sci.* **2016**, *17*, 161. doi:10.3390/ijms17020161.
40. Wang, G.; Wang, T.; Jia, Z.H.; Xuan, Z.P.; Pan, D.L.; Guo, Z.R.; Zhang, J.Y. Genome-wide bioinformatics analysis of *MAPK* gene family in kiwifruit (*Actinidia chinensis*). *Int. J. Mol. Sci.* **2018**, *19*, 2510. doi:10.3390/ijms19092510.
41. Li, J.B.; Zhang, J.; Jia, H.X.; Yue, Z.Q.; Lu, M.Z.; Xin, X.B.; Hu, J.J. Genome-wide characterization of the *sHsp* gene family in *Salix suchowensis* reveals its functions under different abiotic stresses. *Int. J. Mol. Sci.* **2018**, *19*, 3246. doi:10.3390/ijms19103246.
42. Wang, P.; Heitman, J. The cyclophilins. *Genome Biol*. **2005**, *6*, 226. doi:10.1186/gb-2005-6-7-226.
43. Cutler, S.R.; Ehrhardt, D.W.; Griffitts, J.S.; Somerville, C.R. Random GFP::cDNA fusions enable visualization of subcellular structures in cells of *Arabidopsis* at a high frequency. *PNAS* **2000**, *97*, 3718–3723. doi:10.1073/pnas.97.7.3718.
44. Ying, S.H.; Feng, M.G. Novel blastospore-based transformation system for integration of phosphinothricin resistance and green fluorescence protein genes into *Beauveria bassiana*. *Appl*. *Microbiol*. *Biotechnol.* **2006**, *72*, 206–210. doi:10.1007/s00253-006-0447-x.

| 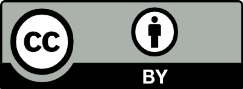 | © 2019 by the authors. Submitted for possible open access publication under the terms and conditions of the Creative Commons Attribution (CC BY) license (<http://creativecommons.org/licenses/by/4.0/>). |
| --- | --- |
